# Characteristics of Gas Permeation Behaviour in Multilayer Thin Film Composite Membranes for CO_2_ Separation

**DOI:** 10.3390/membranes9020022

**Published:** 2019-02-01

**Authors:** Jelena Lillepärg, Sabrina Breitenkamp, Sergey Shishatskiy, Jan Pohlmann, Jan Wind, Carsten Scholles, Torsten Brinkmann

**Affiliations:** 1 Helmholtz-Zentrum Geesthacht, Institute of Polymer Research, Max-Planck-Str. 1, 21502 Geesthacht, Germany; breitenkamp@ressourcen-effizienz.pro (S.B.); sergey.shishatskiy@hzg.de (S.S.); jan.pohlmann@hzg.de (J.P.); jan.wind@hzg.de (J.W.); carsten.scholles@hzg.de (C.S.); torsten.brinkmann@hzg.de (T.B.); 2 Ingenieurbüro Dr. Breitenkamp, Hansastr. 45, 32257 Bünde, Germany

**Keywords:** gas separation, thin film composite membrane, resistance model, dusty gas model, free volume model

## Abstract

Porous, porous/gutter layer and porous/gutter layer/selective layer types of membranes were investigated for their gas transport properties in order to derive an improved description of the transport performance of thin film composite membranes (TFCM). A model describing the individual contributions of the different layers’ mass transfer resistances was developed. The proposed method allows for the prediction of permeation behaviour with standard deviations (SD) up to 10%. The porous support structures were described using the Dusty Gas Model (based on the Maxwell–Stefan multicomponent mass transfer approach) whilst the permeation in the dense gutter and separation layers was described by applicable models such as the Free-Volume model, using parameters derived from single gas time lag measurements. The model also accounts for the thermal expansion of the dense layers at pressure differences below 100 kPa. Using the model, the thickness of a silicone-based gutter layer was calculated from permeation measurements. The resulting value differed by a maximum of 30 nm to the thickness determined by scanning electron microscopy.

## 1. Introduction

The development of a membrane production technology is a key element for transferring the potential of novel, high performance membrane materials into technical application. A thin film composite membrane for gas separation can contain several layers with different permeation properties. The layer thicknesses of multilayer membranes differ from tens of micrometres for the support layers, to tens of nanometres for the ultrathin separation layers. During the penetration through the membrane, the gas molecules interact with all layers of the membrane, where the gas transport characteristics can differ by several orders of magnitude. Thereby, for thin film composite membranes (TFCM) not only the thickness of the separation layer, but also the layer’s interaction with its adjoining layers affects the total membrane performance. For the design of new high-performance gas separation membranes with transport characteristics dominantly governed by the properties of the selective layer material, the accurate prognosis of the gas transport parameters of the entire multilayer structure of the membrane is necessary.

Flat sheet, multi-layer, TFCMs are an ideal means to exploit the toolbox of modern membrane materials and allow for the application of the selected material in form of an extremely thin, dense gas separation layer.

In 1980, Henis and Tripodi presented a mathematical model describing the gas flow through a membrane consisting of different materials arranged in different layers or within one layer as the flow of an electrical current through a network of electrical resistances representing the materials and their arrangement as an analogy to an electrical circuit [1]. The model takes the anisotropy and pressure difference across the TFCM into account and describes the relationship between layers with different morphology (porous and dense) having occluding contact. For two-layer composite membranes the Wheatstone bridge model was also applied [2].

In 1996, an extended model was introduced by Shilton, describing the gas transport mechanism through porous and non-porous layers of hollow fibre membranes via a combination of the Henis and Tripodi resistance model with Knudsen diffusion and viscous flow [3]. The resistance model was applied to the polysulfone (porous) and silicone (dense) layers of a hollow fibre membrane, where the thickness of the dense layer was about 400 nm [4]. The polysulfone/silicone membrane performance was simulated utilising both, the resistance model and the boundary layer theory for description of the concentration polarisation depending on the flow rate and pressure difference [5]. With the resistance in series model permeance and selectivity for the defect-free composite membrane can be effectively predicted [6].

It is apparent that the value of the permeance of a TFCM is smaller than the intrinsic permeance of its selective layer. This fact indicates that the resistance of the porous support layer is not negligible. Evidently, the parameters of the porous support structure such as pore diameter, pore size distribution and tortuosity significantly influence the resulting membrane characteristics, an important fact for the application of modern polymeric membrane materials as polyacetylenes [7], polymers of intrinsic microporosity (PIMs) [8,9], blockcopolymers for CO_2_ separation [10] or thermally rearranged polymers having extremely good gas transport characteristics [11]. Hence, simulations of mass transfer processes through non-porous dense layers deposited on porous support layers were conducted recently [12,13,14]. The effects of pore size and pore distribution were taken into account in these studies.

The equipment available at Helmholtz-Zentrum Geesthacht (HZG) allows for the manufacture of membranes with extremely thin selective separation layers and the measurement of gas transport properties with high accuracy [15]. In the scope of this work, a series of measurements was carried out in order to include the contribution of each layer to the overall resistance into one model.

This work focuses on the modeling of the permeation behaviour in multilayer TFCM as a function of temperature. The porous support structures were described using the Dusty Gas Model (DGM) developed by [16] and implemented for porous support structures by Breitenkamp (neé Kipp) [17]. The permeation in the dense gutter layer and in the separation layer was considered, using parameters derived from single gas time-lag measurements.

## 2. Materials and Methods

### 2.1. Materials and Preparation of Samples

PolyActive^TM^ 1500 (further PolyActive^TM^) in a pellet form was received from PolyVation BV, the Netherlands.

PolyActive^TM^ and polydimethylsiloxane (PDMS) (The suppliers and characteristics of the PDMS and PE non-woven cannot be disclosed due to licensing limitations.) thick films having a thickness more than 100 µm were casted from a 3 wt.% polymer solution in tetrahydrofuran and isooctane (both of Th. Geyer, Germany), respectively as described in [18].

HZG developed multilayer thin film composite membranes generally consist of support, gutter, thin selective, and protection layers. The polyester (PE) non-woven support is coated with a porous poly(acrylonitrile) (PAN) layer by means of a phase inversion process [19]. The dense PDMS gutter layer ensuring efficient passage of gases penetrating through the selective layer to the pores of the support is deposited onto porous support so that PDMS does not penetrate into pores, and also then forms a continuous film of 100 nm to 200 nm thickness. The selective layer, in the case of the present study PolyActive™, is deposited on top of the PDMS gutter layer. Both PDMS and PolyActive™ are applied on a pilot scale membrane coating machine available at HZG [20,21,22,23].

### 2.2. Membrane Characterization

The thickness of isotropic PDMS and PolyActive™ thick films was measured with a digital micrometer DELTASCOPE^®^ FMP10 (Fischer, Germany).

The morphology of the porous PAN membrane and TFCMs based upon it was studied by analysis of electron microscopy micrographs obtained on LEO Gemini 1550 VP and Merlin (both Zeiss, Germany) scanning electron microscopes. Samples for cross-sectional micrographs were prepared by breaking the membrane in liquid nitrogen. Samples for cross-sectional and surface analysis were fixed on a sample holder with a conductive paste before they were sputtered with approximately 2 nm thick platinum coating. The surface porosity and pore diameters of PAN were determined using Image Management System (IMS) software (Imagic, Switzerland).

The gas transport properties of the support structure were investigated for the pure gases of hydrogen, methane, nitrogen, oxygen and carbon dioxide. The membrane sample consisting of non-woven polyester and a PAN layer was mounted into a test cell having a diameter of 47 mm. The temperature in the cell was kept constant at about 25 °C using a thermostated water bath. The transmembrane pressure difference using 3051 Pressure transmitter Rosemount^®^ (Emerson, Germany), feed pressure using digital manometer LEO 2 KELLER (Keller, Switzerland) and volumetric flow rate through the membrane using primary flow meter Definer 220 (Mesa Laboratories, USA) were acquired. The following feed pressure ranges were investigated for each gas: 250 kPa to 252 kPa, 370 kPa to 372 kPa and 500 kPa to 502 kPa. Measurements of the volumetric flow rate ${\dot{V}}_{P,i}$ of a single gas i at transmembrane pressure differences ranging from 1 kPa to 60 kPa were performed in turn for each of the aforementioned feed pressure ranges. The use of pressures instead of fugacities is justified since the absolute pressures involved are sufficiently low.

Gas transport properties of thick isotropic films were characterised by a “time-lag” experiment implementing a constant volume, variable pressure method using an in-house developed measurement instrument as described elsewhere [24]. The single gas permeability of each sample was determined in the temperature range from 20 to 80 °C. Each measurement was repeated at least 3 times at feed pressures of (64, 50, 40, 30) kPa. The permeability coefficient *P* (mol·m·m^−2^·s^−1^·Pa^−1^) of a single gas *i* was determined as:

(1) $$P_{i}\;=\frac{V_{P,i}\;\cdot \delta \;\cdot {(p}_{P2\;}-{\;p}_{P1})}{A\;\cdot \;R\;\cdot \;T\;\cdot \;\Delta t\;\cdot \;\left(p_{F\;}-\frac{p_{P2\;}{\;+\;p}_{P1}}{2}\right)},$$

where *V_P,i_* is the constant permeate volume (m^3^), *δ* is the film thickness (m), *A* is the effective area of membrane (m^2^), *R* is the gas constant (8.314 Pa·m^3^·mol^−1^·K^−1^), *T* is the temperature (K), *p_F_* is the feed pressure, *p_P_*_1_ and *p_P_*_2_ are the permeate pressures (Pa) at the start and at the end of the pressure increase, respectively, and ∆*t* is the time for permeate pressure increase from *p_P_*_1_ to *p_P_*_2_ (s).

The gas permeances for the TFCMs were measured using the pressure increase facility, designed and built at HZG [25], where the aforementioned constant volume, variable pressure method is realized [26]. Single gas permeation data were determined at (50, 75, 100) kPa feed pressures and in the temperature range from 20 °C to 80 °C.

The permeance *L* (mol m^−2^·s^−1^·Pa^−1^) of each layer for the gas *i* can be calculated employing the following equation for a TFCM consisting of a porous support and one or more dense layers:

(2) $$L_{i}\;=\;\frac{V_{P,i}\;}{A\;\cdot \;R\;\cdot \;T\;\cdot \;\Delta t}ln\left(\frac{p_{F}\;{-\;p}_{0}}{p_{F}\;{-\;p}_{P\left(t\right)}}\right),$$

where *p_F_*, *p*_0_, and *p_P_*_(*t*)_ (Pa) are the pressures of the feed, of the permeate side at the beginning, and of the permeate side at the end of measurement, respectively, and Δ*t* (s) is the time of the measurement between permeate pressures *p*_0_ and *p_P_*_(*t*)_.

The permeance of component *i* in layer *j* is calculated by [27]:

(3) $$L_{j,i\;}=\;\frac{P_{j,i}}{\delta_{j,i}}.$$

The thickness of the layer is assumed to be dependent on the penetrating gas *i*, since different gases will cause different degrees of swelling.

Two sets of circular membrane samples (called Stamps in the following) were prepared. Their designations are given in Table 1.

## 3. Model Development

For the mathematical description of mass transport in multilayer composite membranes, various models can be used. Since the mass transport in porous membranes is based on a mechanism different from those in dense membranes, the two membrane types are considered separately.

### 3.1. Gas Transport in Porous Layers

**Figure 1:**
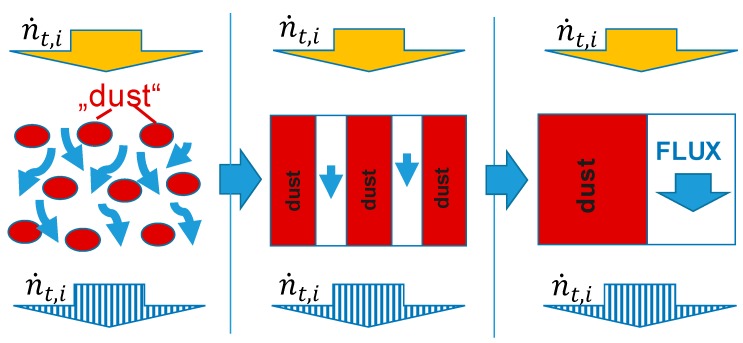
Schematic diagram of the modeled thin film composite membranes (TFCM): porous support as equivalent of parallel slabs.

A membrane consisting of a non-woven polyester and a PAN porous layer was used to experimentally study and simulate the transport processes occurring in the TFCM support structure. The DGM developed by Mason and Malinauskas [16] and described in detail by Krishna and Wesslingh [28] was employed in this paper. The DGM describes the pore wall as large motionless “dust” molecules that are uniformly distributed in space (Figure 1).

This model takes Knudsen diffusion, molecular diffusion in case of multicomponent mixtures and convective flow including the porous medium effect into account and is based on the Maxwell–Stefan diffusion equation [29]. The linear form of the DGM for single gas transport through the porous layer is:

(4) $${\overset{\cdot }{n}}_{PS,i}^{”}=\;\frac{1}{R\cdot T}\;\cdot \;\frac{\varepsilon }{\tau }\;\cdot \;\frac{\Delta p_{i}}{\delta_{PS}}\;\cdot \;\left(\frac{4}{3}\;\cdot d_{pore}\;\cdot \;\sqrt{\frac{R\cdot \;T}{2\cdot \;\pi \;\cdot \;M_{i}}}\;+\;\frac{d_{pore}^{2}\;\cdot {\;p}_{a,i}}{32\;\cdot {\;\eta }_{i}}\right),$$

where:

(5) $$p_{a,i}\;=\;0.5\;\cdot \left(p_{F,i\;}{\;+\;p}_{P,i}\right),$$

where ${\overset{\cdot }{n}}_{PS,i}^{”}$ is the molar flux through the membrane divided by area (mol·m^−2^·s^−1^), *ε* is the accessible fractional void volume of the porous medium, *τ* is the tortuosity factor characterising the porous matrix, Δ*p_i_* is the difference of pressure between feed *p_F,i_* and permeate side *p_P,i_* of the layer (Pa), *δ_ps_* is the thickness of the porous support (m), *d_pore_* is the pore diameter (m), *M_i_* is the molecular weight of gas (kg·mol^−1^), *p_a,i_* is the average pressure (Pa) and *η_i_* is the dynamic viscosity of the gas (Pa·s).

**Figure 2:**
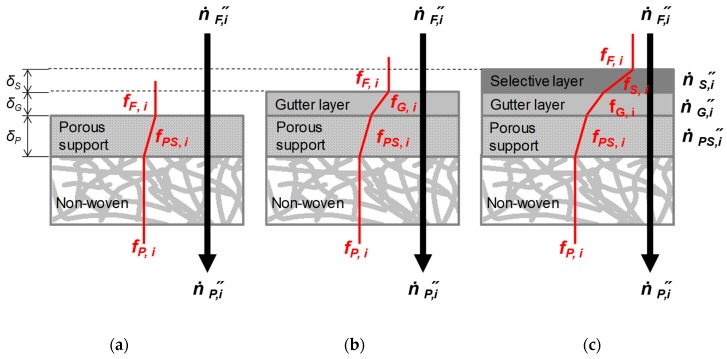
Scheme of modelled thin film composite membranes (TFCM): porous support, corresponding to Stamp 1 and 2 (**a**); porous support with one dense layer (gutter layer, corresponding to Stamp 1-2 and 2-2) (**b**); porous support with two dense layers: gutter and selective layers, corresponding to Stamp 1-3 and 2-3 (**c**).

The non-woven supporting layer is not included into the DGM calculation because the mass transfer resistance of the non-woven can be assumed to be negligible due to extremely open structure formed by the polymer fibres (Figure 2a).

### 3.2. Gas Permeation in Dense Layers

Generally, the mass transfer of a single component through a dense polymer membrane can be described as a function of temperature and pressure. The following Equation (6) of the Free Volume model (FVM) is applied to dense polymers in which the flux of a penetrant can be described by Fick’s first law and its sorption behaviour expressed by Henry’s law [30].

(6) $$P_{i\;}{\;=\;P}_{\infty ,i}^{0}\;\cdot \;exp\left(-\;\frac{E_{act,\;i}}{R\;\cdot \;T}{\;+\;m}_{0,\;i\;}\cdot {\;p}_{a,i}\;\cdot {\;exp\;(m}_{T,i\;}\cdot \;T)\right),$$

where $P_{\;\infty ,i}^{0}$ is the permeability for infinite temperature and the pressure approaching zero, *E_act,i_* is the activation energy of permeability (J·mol^−1^), *m_0,i_* is the swelling factor at zero temperature (Pa^−1^) and *m_T,i_* takes the temperature dependency of swelling into account (K^−1^). The FVM can also be applied to dense layers of a TFCM:

(7) $$L_{i\;}{\;=\;L}_{\infty ,i}^{0}\;\cdot \;exp\left(-\;\frac{E_{act,\;i}}{R\;\cdot \;T}{\;+\;m}_{0,\;i\;}\cdot {\;p}_{a,i}\;\cdot {\;exp\;(m}_{T,i\;}\cdot \;T)\right),$$

where

(8) $$L_{\infty ,i}^{0}\;=\;\frac{P_{\infty ,i}^{0}}{\delta },$$

is in compliance with Equation (3) where the activation energy and swelling parameters are theoretically identical to Equation (6) for amorphous, rubbery polymers.

### 3.3. Resistance Model for TFCM

The driving forces for the permeation of a gas *i* through the polymeric membrane can be estimated as the difference of partial pressures between the feed and the permeate sides of the membrane Δ*p_i_* at conditions where the ideal gas law can be assumed to be valid. In this case molar flux of gas *i* through the unit of membrane area can be expressed as:

(9) $${\overset{\cdot }{n}}_{i}^{”}\;=L_{i}\cdot \Delta p_{i}.$$

In case a multilayer composite membrane is considered, Equation (9) is valid for each of the membrane layers *j*:

(10) $${\overset{\cdot }{n}}_{j,i}^{”}\;=L_{j,i}\cdot \Delta p_{j,i}$$

where Δ*p_j_,_i_* is assumed to contain hypothetical partial pressures in-between layers (*cf.* Figure 2, where the fugacities shown are to be replaced by partial pressure) if neither feed nor permeate partial pressures are involved.

Due to the continuity equation, the overall flow rate ${\overset{\cdot }{n}}_{t,i}$ must be constant throughout all the layers *j = 1, 2,…, n_layers_*.

(11) $${\overset{\cdot }{n}}_{t,i}={\overset{\cdot }{n}}_{PS,i}={\overset{\cdot }{n}}_{G,i}={\overset{\cdot }{n}}_{S,i}=\ldots ={\overset{\cdot }{n}}_{n_{layers,i}}.$$

**Figure 3:**
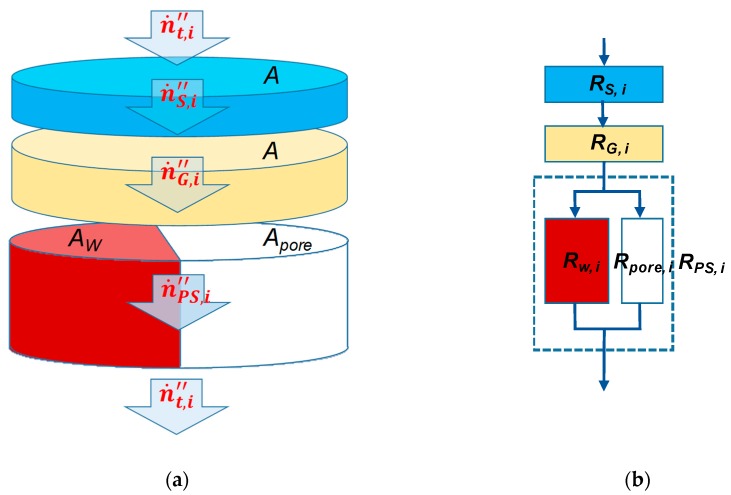
Schematic diagram of the modeled TFCM: flow rate trough multilayer membrane (**a**); TFCM as analogy to the electric circuit (**b**). Membrane area *A* is divided in *A_W_* for pore wall and *A_pore_* for pores, with respective resistances *R_W_* and *R_pore_*.

Writing the Equation (11) in terms of fluxes for the membrane area *A* gives [16] (Figure 3a):

(12) $${\overset{\cdot }{n}}_{j,i}^{”}={\overset{\cdot }{n}}_{PS,i}^{”}={\overset{\cdot }{n}}_{G,i}^{”}={\overset{\cdot }{n}}_{S,i}^{”}=\ldots ={\overset{\cdot }{n}}_{n_{layers,i}}^{”},$$

Thus, the total flux across the porous support is related to the flux ${\overset{\cdot }{n}}_{pore,i}^{”}$ in the pore as:

(13) $${\overset{\cdot }{n}}_{t,i}^{”}={\overset{\cdot }{n}}_{PS,i}^{”}=\frac{A_{pore}}{A}\cdot {\overset{\cdot }{n}}_{pore,i}^{”}$$

where $A_{pore}$ is the area of the porous region.

The flow rate can be expressed as function of a resistance to flow as proposed by Henis and Tripodi in analogy to an electric circuit [1]. The resistance model determines the total partial pressure drop of the gas *i* across the membrane as the sum of the individual partial pressure drops across the layers *j* of a TFCM. The resistance to permeate flow *R_j,i_* was defined as equivalent to the electrical resistance:

(14) $$R_{j,i}\;=\;\frac{\delta_{j}}{P_{j,i}\cdot A}.$$

For the composite membrane (Figure 3b) the total resistance *R_t,i_* in this work is determined from the resistance-in-series model [5]. We take into account the resistance of the porous support *R_PS,i_*, the resistance of the gutter layer *R_G,i_* and the resistance of the selective layer *R_S,i_*. For the simplicity of the resistance model of the porous medium, we assume that pores are homogeneous through the thickness of the porous support. The porous medium consists of a number of non-interconnected circular capillaries with diameter *d_pore_*. The total resistance of a multilayer membrane as shown in Figure 3b can be expressed as:

(15) $$R_{t,\;i}\;=\;\sum_{j=1}^{n_{layers}}R_{j,i}{\;=\;R}_{PS,i}{\;+\;R}_{G,i}{\;+\;R}_{S,i}.$$

where the resistance *R_PS,i_* was determined analogous of parallel electrical circuit:

(16) $$R_{PS,\;i}\;=\;\frac{R_{w}\;\cdot {\;R}_{pore}}{R_{w}{\;+\;R}_{pore}}\;\approx {\;R}_{pore}$$

The resistance *R_W_* and *R_pore_* are resistances of impermeable bulk substrate (pore wall) and permeable pores, respectively.

This approach allows for the description of the TFCM by using the permeation characteristics measured separately for the individual building blocks of the membrane.

## 4. Results and Discussion

The validity of the model developed above was tested by comparing the layer thickness determined from scanning electron microscope (SEM) micrographs and the thicknesses determined from gas transport parameters of thick films and TFCMs.

### 4.1. Application of DGM to Experimental Data

The permeation of two stamps of a porous PAN membrane was investigated at varying feed and permeate pressure differences and at different temperatures as described above. Stamps (Stamp 1 and Stamp 2) were taken from two different batches of membrane where the non-woven was coated with a layer of porous PAN using the same recipe. The experimental gas transport data was obtained for gases with different molecular weights: H_2_, CH_4_, N_2_, O_2_ and CO_2_.

The molar flux may be expressed as:

(17) $${\overset{\cdot }{n}}_{i}^{”}=\frac{1}{A}\cdot \frac{{\overset{\cdot }{V}}_{i}^{N}p^{\emptyset }}{R\cdot T^{\emptyset }},$$

where is ${\overset{\cdot }{V}}_{i}^{N}$ the volumetric flow rate of gas *i* at normal pressure *p^Ø^* = 101.3 kPa and temperature *T^Ø^* = 0 °C, respectively.

The use of Equation (17) for the molar flux in Equation (4) and expressing the resulting equation in linear form yield:

(18) $$\underset{Y}{\underbrace{\frac{{\overset{\cdot }{n}}_{i}^{”}\cdot \;\sqrt{M_{i}\;\cdot \;R\;\cdot \;T}}{\Delta p_{i}}}}\;=\frac{C_{0}}{\delta_{PS}}+\frac{C_{1}}{\delta_{PS}^{2}}\;\cdot \;\underset{X}{\underbrace{\frac{p_{i,a}\;\cdot \delta_{PS}}{\eta_{i}}\;\cdot \;\sqrt{\frac{M_{i}}{R\cdot T}}}},$$

where

(19) $$C_{0}\;=\;\frac{4\;\cdot {\;d}_{pore\;}\;\cdot \;\varepsilon }{3\;\cdot \;\tau \;\cdot \;\sqrt{\;2\;\cdot \;\pi }}\;\mathrm{and}\;C_{1}\;=\;\frac{d_{pore}^{2}\;\cdot \;\varepsilon }{32\;\cdot \;\tau }.$$

**Figure 4:**
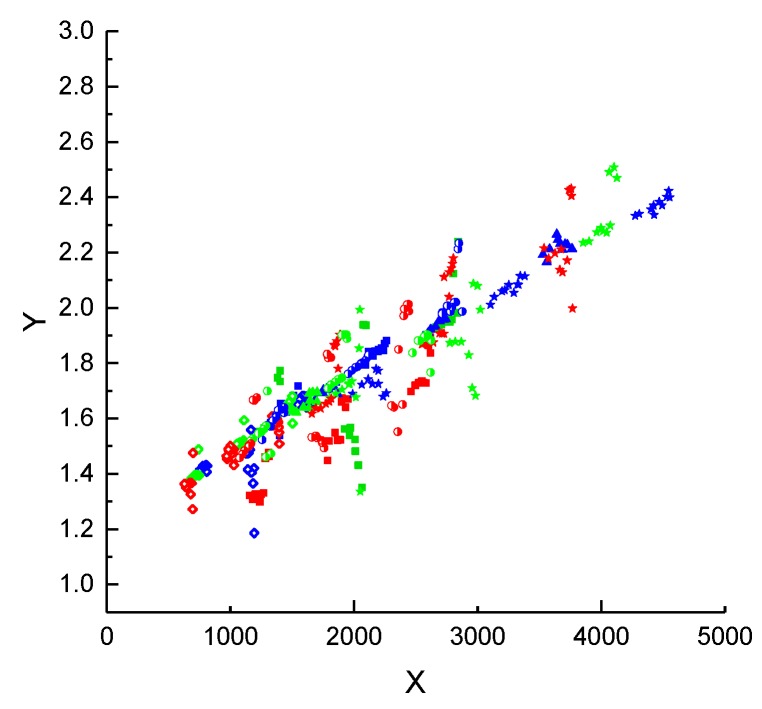
Correlation of $Y=1\cdot 10^{-4}\frac{{\dot{n}}_{i}^{”}\cdot \sqrt{M_{i}\cdot R\cdot T}}{\Delta p}$ and $\;X=\frac{p_{i,a}\cdot \delta }{\eta_{i}}\cdot \sqrt{\frac{M_{i}}{R\cdot T}}$ values for porous support (Stamp 1). ◇ H_2_, ▲ CH_4_, ■ N_2_, ◗ O_2_, 🟊 CO_2_. The blue, green and red colors reflect the temperatures of (30, 50, 70) °C respectively.

**Figure A1:**
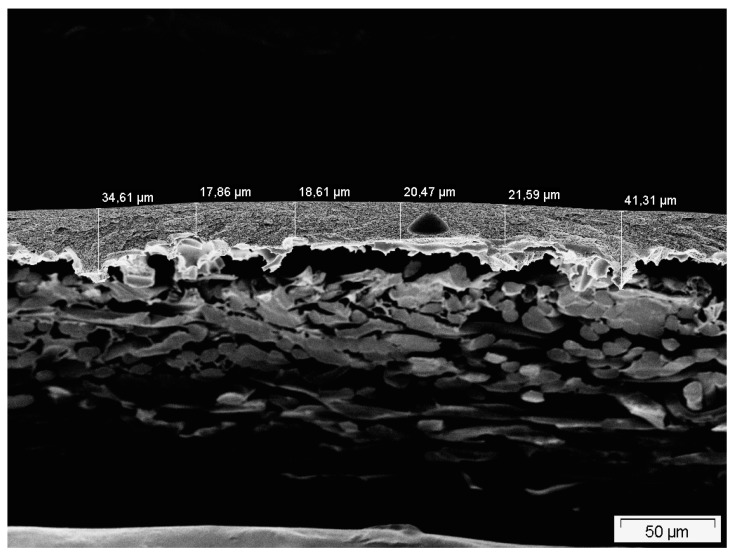
Scanning electron micrographs of poly(acrylonitrile) (PAN) support on the non-woven polyester (Stamp 2).

Calculating the X and Y values from the experimental data of the single gas measurements and plotting them as shown in Figure 4 allowed for determination of of *C*_0_*/*$\delta_{PS}$ and *C*_1_*/*$\delta_{PS}^{2}$ as linear regression’s Y-intercept and the slope, respectively. The thickness *δ_PS_* of the porous PAN layer for the composite membrane was obtained from SEM micrographs (Appendix A, Figure A1) and estimated to be 30 µm ± 7 µm. The error of the porous layer thickness determination is likely to originate from the roughness of the polyester non-woven.

**Figure 5:**
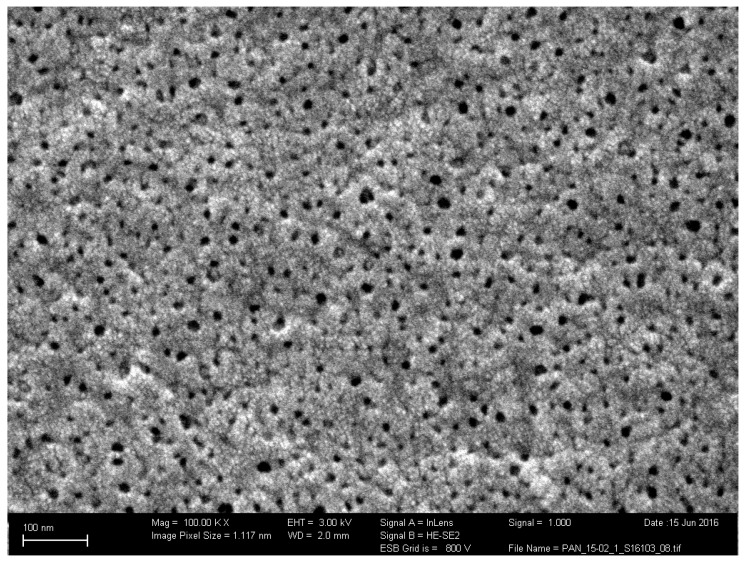
Scanning electron microscope (SEM) micrograph of the poly(acrylonitrile) (PAN) surface (Stamp 1). The maximum visible pore diameter is 20 nm.

**Figure A2:**
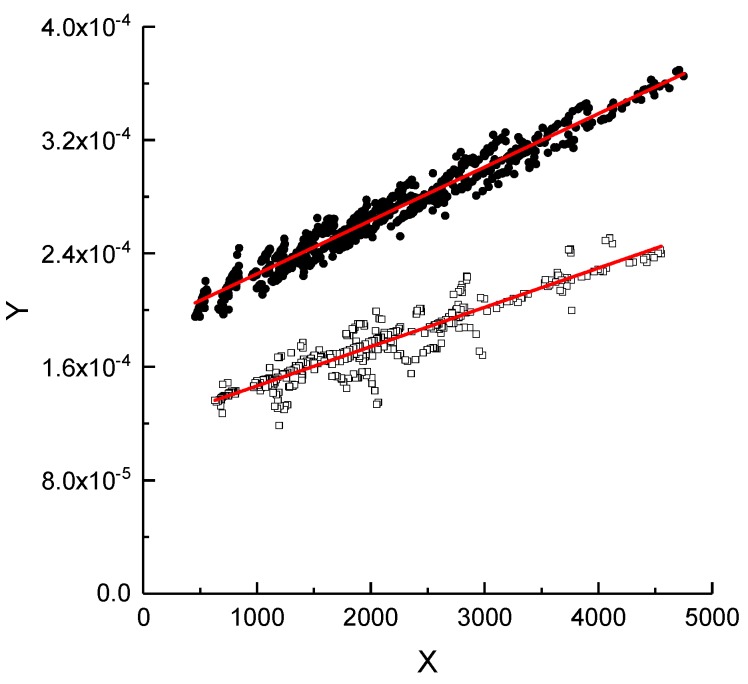
Data plotting of *Y* values as a function of *X*. Where $Y\;=\;\;\frac{{\overset{\cdot }{n}}_{i}^{”}\cdot \;\sqrt{{M\;}_{i}\cdot R\cdot T}}{\Delta p}$ and $X\;=\;\frac{p_{i,a}\;\cdot {\;\delta }_{ps}}{\eta_{i}}\;\cdot \;\sqrt{\frac{M_{i}}{R\;\cdot \;T}}$ [□ Stamp 1, ● Stamp 2].

The values of the *ε/τ* and the average pore size *d_pore_* for stamp Stamp 1 were calculated from Equations (18) and (19) as 0.056 and 133 nm, respectively. The measurements with the Stamp 2 (Appendix A, Figure A2) taken from another PAN membrane batch gave *ε/τ* and *d_pore_* values of 0.099 and 119 nm, respectively. The pore diameter visible on the SEM micrograph of the PAN membrane surface (Figure 5) had a maximum value of 20 nm. This value is six times smaller than the pore size calculated via the DGM. The spongy, asymmetric morphology of the PAN membrane is characterized by high irregularity of the porous structure across the membrane thickness, with pores tapering towards a smaller diameter as they approach the upper, feed side surface of the membrane [20]. Hence, it can be assumed that the pore size values determined using Equation (18) represent an average value for the entire support structure.

**Figure 6:**
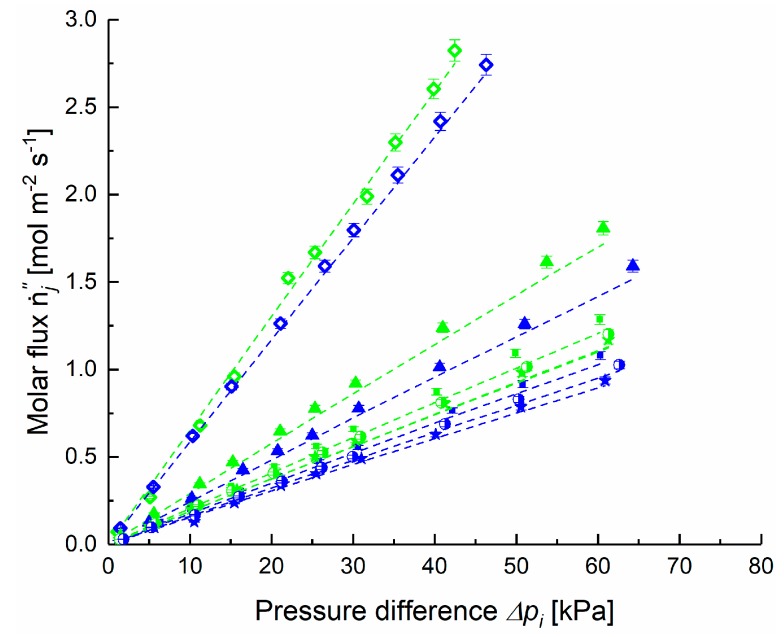
Measured [◇ H_2_, ▲ CH_4_, ■ N_2_, ◗ O_2_, 🟊 CO_2_] and calculated via Dusty Gas Model (DGM) (dashed lines) molar fluxes ${\overset{\cdot }{n}}_{i}^{”}$ through the porous support (Stamp 1) in dependence on pressure difference. Green colors: at 30 °C, blue at 50 °C.

Based on these results for the PAN porous layer, the first experiment-model comparisons are given in Figure 6 where the single component permeate flux ${\overset{\cdot }{n}}_{i}^{”}$ is plotted as a function of the pressure difference across the membrane Δ*p_i_*. The permeate flux for all gases increases with an increase in pressure difference. The slope of pressure dependence follows the order of molecular weights of gases studied: H_2_, CH_4_, N_2_, O_2_, and CO_2_. Hydrogen, for example has the smallest molecular weight and shows the largest permeate flux. The DGM predicts that the molar flux ratio depends on the molecular weight of the gases because of the diffusive component of mass transfer stemming from molecule-pore wall collisions, i.e., Knudsen diffusion [31]. Thus, the principle of representing the pore wall as consisting of “giant” molecules in the DGM appears to be well suited to represent the gas transport in porous support layers [27].

### 4.2. Estimation of FVM Parameters for Dense Layers

The parameters for the FVM may be determined directly by using Equations (6) and (7) for the entire composite membrane, as described in e.g., [32]. For a more accurate prediction of the permeation behaviour of TFCM, the individual layers made from different polymers with different thicknesses have to be considered individually and the FVM parameters have to be determined for each layer by using Equations (6)–(8).

**Figure 7:**
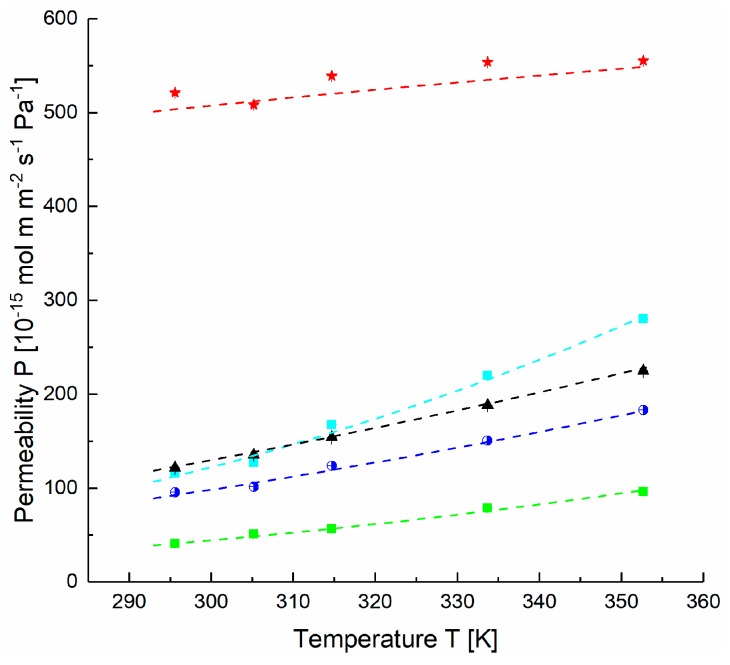
Permeability coefficients of gases in polydimethylsiloxane (PDMS) [◇ H_2_, ▲ CH_4_, ■ N_2_, ◗ O_2_, 🟊 CO_2_] determined by the time-lag method and modeled with free volume model (FVM) (dashed lines).

A thick film sample of PDMS was prepared and gas transport properties were determined using the time-lag method. The temperature dependence of the permeability coefficients for all gases is presented in Figure 7 and served for estimation of the activation energies and the permeabilities at infinite temperature for the investigated gases. The values presented in Table 2 are in agreement with the data reported in previous investigations [33,34,35]. At the operation conditions employed in this study, swelling was not assessed and the respective parameters of the FVM were therefore not determined. The data shown for the CO_2_ selective block copolymer PolyActive™ also listed in Table 2 were determined as described above for PDMS using a thick film sample.

The swelling effect of CO_2_ on the pure PDMS at pressures up to 1 MPa was reported to have an effect of less than 5% on the thickness [36,37,38]. In this work we assume that the swelling effect of gases on the single layer can be neglected in the investigated pressure range up to 60 kPa [39]. Hence, the FVM (Equations (6) and (7)) simplifies to be an Arrhenius relationship.

From solving Equations (3) to (16) for the porous support and gutter layer, the thickness of the gutter layer *δ_G_* can be estimated. If the tortuosity factor is known, the porosity $\frac{A_{pore}}{A}$ can be determined. The geometrical definition of tortuosity implies that it is always larger than unity [40]. In our study, we estimated *τ* to be in the range from 1.6 to 5 in accordance with the guidelines developed for porous media [41].

In the following section, four different scenarios will be defined for calculating the thickness of the gutter layer from the permeation experiments as described in Section 3 by Equations (3) to (16). The scenarios were:

1. Scenario 1: using gas transport data of Stamp 1-2 and estimated pore diameter *d_pore_* = 133 nm and structure parameter *ε/τ=0.056*. Thickness of porous support *δ_PS_* = 30 µm.
2. Scenario 2: using gas transport data of Stamp 1-2 with *d_pore_* = 133 nm and *ε/τ = 0.056*. The thickness of the porous support *δ_PS_* was decreased from 30 µm to 23 µm in order to examine the influence of the roughness of the porous support.
3. Scenario 3: using gas transport data of Stamp 1-2 with *d_pore_* = 133 nm and *ε/τ = 0.056*. The thickness of the porous support *δ_PS_* was increased from 30 µm to 40 µm.
4. Scenario 4: using gas transport data of Stamp 2-2 with *d_pore_* = 119 nm and *ε/τ = 0.099* in order to compare the results of modeling with the results for Stamp 1.

**Figure 8:**
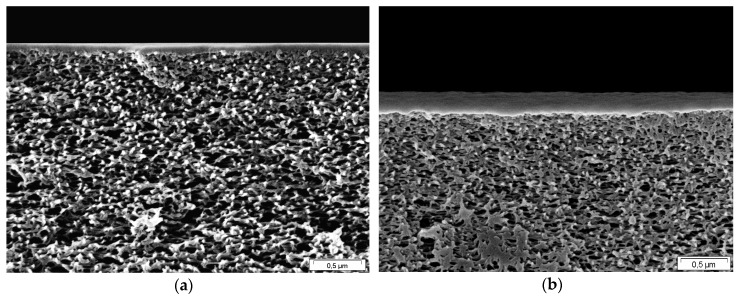
SEM micrographs of Stamp 1-2 (**a**) and Stamp 2-2 (**b**).

The thickness of the gutter layer estimated from gas permeation measurements according to Equations (3) to (16) for the studied gases corresponds well to the thickness values determined from SEM micrographs [42] as shown in Table 3: 105 nm for Stamp 1-2 and 150 nm for Stamp 2-2 (Figure 8a,b respectively). If the uncertainty of the porous layer thickness will be taken into account and the thickness *δ_ps_* will be reduced by 25% (Scenario 2) the average thickness of the gutter layer *δ_G_* will, according to our model, increase from 140 to 150 nm. Since the DGM does not take into account the pore size distribution, the geometric parameter *ε/τ* of the porous support is causing the variation of the thickness *δ_ps_* in Scenario 3. The SD value of the gutter layer thicknesses determined considering transport data of different gases reaches 65 nm for Stamp 1-2.

**Figure 9:**
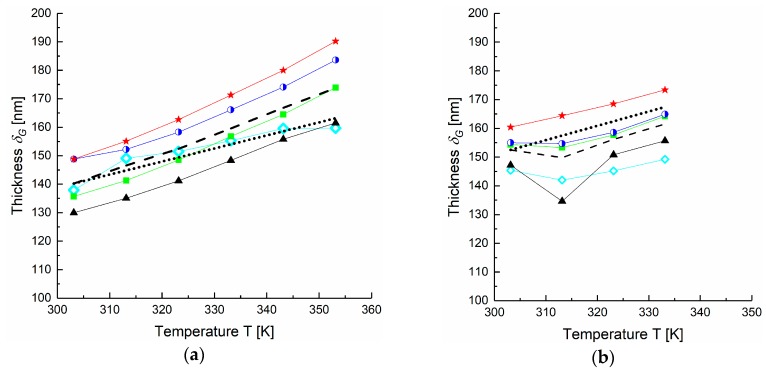
Numerically estimated thicknesses of PDMS gutter layer for Stamps 1-2 (**a**) and 2-2 (**b**) [◇ H_2_, ▲ CH_4_, ■ N_2_, ◗ O_2_, 🟊 CO_2_]. Dashed lines on both plots show average value of the gutter layer thickness. Dotted lines reflect the expected thickness change due to thermal expansion Δ*α* = 10.9 × 10^−4^ K^−1^.

**Figure 10:**
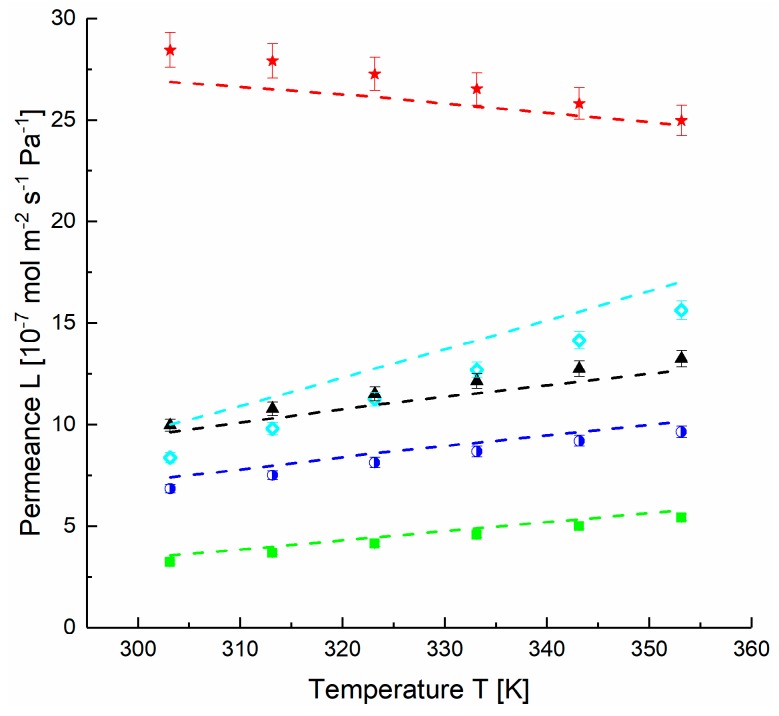
Measured [◇ H_2_, ▲ CH_4_, ■ N_2_, ◗ O_2_, 🟊 CO_2_] and modeled (dashed lines) using Equations (4) to (14) and 17 permeances for Stamp 1-2. Data of TFCM with two layers: porous support and gutter layer. Scenario 1.

Figure 9 shows the temperature dependence of the calculated gutter layer thickness for both Stamps 1-2 and 2-2 using the parameters of Scenario 3 and 4, respectively. The change of thickness with the temperature for both membrane samples correlates with the PDMS thermal expansion coefficient Δ*α* = 10.9 × 10^−4^ K^−1^ reported in [43]. The thermal expansion coefficients estimated from our experiments as (*dV/dT)/V* using the assumptions of Scenario 1 and 4 are 15.9 × 10^−4^ K^−1^ and 3.9 × 10^−4^ K^−1^ for Stamp 1-2 and Stamp 2-2, respectively, the values are in the same order of magnitude as the tabulated one. The thermal expansion coefficient was applied to simulate the gas permeances of the TFCM consisting of a porous and a gutter layer (Figure 10). The permeances modeled with the calculated average thickness *δ_G_* = 140 nm at 30 °C and Δ*α* = 10.9 × 10^−4^ K^−1^ for PDMS gutter layer give less than 10% discrepancy between measured and prognosis values.

The parameters of the porous layer e.g., *ε/τ* as well as the interaction of porous and gutter layers should be taken into account to describe the delivery of the gas from the continuous gutter layer into the pores of the porous support, especially in case of highly permeable gases when the permeance of the selective layer is close to the one of the porous layers [44]. The DGM takes into account the molecular weight of the fluid only, but not its molecular volume or shape. The significant difference in the calculated PDMS layer thickness for different gases can reflect the effect of penetrant parameters other than molecular weight on penetration through the composite membrane. The paper [45] shows the correlation of the gas kinetic diameter with the diffusion coefficient.

**Figure 11:**
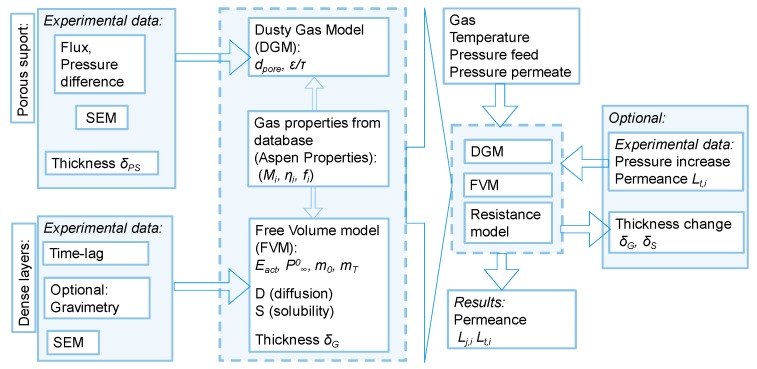
Simplified flowchart of model applied for description of different layer contributions in gas transport through TFCM.

Technically, if the thicknesses of the gutter and support layers are known from SEM investigations, the prognosis of total permeance for the TFCM depending on temperature and at pressures less than 100 kPa can be carried out (Figure 11). If experimental data of permeances are available, the reciprocal estimation of the thicknesses *δ_G_* and *δ_S_* is possible.

Based on the successful modeling of a bilayer (PDMS gutter layer on PAN porous support) membrane it is possible to make a new step in direction of modeling of a more complex membrane consisting of aforementioned layers and one additional layer of PolyActive™. This membrane is widely studied for separation of CO_2_ from e.g., flue gases [10,32] and it is of tremendous importance to develop a model adequately describing the behaviour of the membrane in various environments and various conditions.

**Figure 12:**
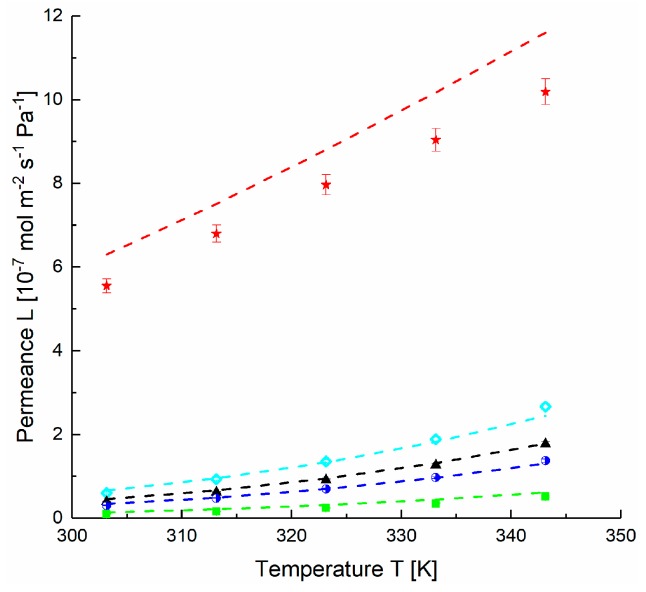
Measured [◇ H_2_, ▲ CH_4_, ■ N_2_, ◗ O_2_, 🟊 CO_2_] and modeled (dashed lines) data of TFCM with two dense layers (Stamp 1-3): porous support, gutter layer (PDMS) and selective layer (PolyActive^TM^). The model parameters are: *ε/τ =* 0.056, *d_pore_* = 133 nm, feed pressure 50 kPa, Δ*α* (PDMS) = 10.9 × 10^−4^ K^−1^, Δ*α* (PolyActive^TM^) = 1.2 × 10^−4^ K^−1^. Thickness parameters *δ_G_* = 130 nm, *δ_S_* = 90 nm are determined by SEM (Appendix A, Figure A3).

The permeances of PolyActive™ TFCM modeled with average thicknesses *δ_G_* = 130 nm and *δ_S_* = 90 nm determined by SEM micrographs for the Stamp 1-3 give less than 10% discrepancy between experimental and prognosis values for fast permeating gases and about 3% for slow permeating gases. Similar behaviour was observed for the Stamp 2-3, with no sudden changes. The thermal expansion coefficients Δ*α* (PDMS) = 10.9 × 10^−4^ K^−1^, Δ*α* (PolyActive^TM^) = 1.2 × 10^−4^ K^−1^ [46] were applied to simulate the gas permeances of the TFCM consisting of porous, gutter layer and the selective layer (Figure 12). Equations (3) to (16) were used in combination with values tabulated in Table 2 for PolyActive^TM^.

The separation layer affects the results as the main contributor in the prognosis of permeance values. For the bilayer or trilayer samples, the coupling between sublayer and toplayer can influence their physical properties [47]. However, taking into account the thermal expansion coefficient prevents the eventual increase in discrepancy between experimental and prognosis permeance values with temperature grow. As this study shows, the estimation of geometrical parameters for the subordinated layers and taking into account their gas transport properties can significantly increase the accuracy of the prognosis for TFCM under changing working conditions.

## 5. Conclusions

Porous, porous/gutter layer and porous/gutter layer/selective layer types of TFCM were investigated for their gas transport properties. A model describing the individual contributions of the different layers’ mass transfer resistances was successfully employed. The porous support structures were described using the Dusty Gas Model whilst the permeation in the dense gutter and separation layers was described by applicable models such as the Free-Volume model, using parameters derived from single gas time lag measurements. The model was employed to calculate the thickness of a silicone-based gutter layer from permeation measurements and compared to the thickness determined by SEM.

The model takes into account the dependence of the total permeance on the properties of the porous layer, as well as the thermal expansion of dense layers at pressures below 100 kPa.

The developed approach allows for the description of gas transport through the multilayer TFCM for variety of gases using performance data of the material that make up the individual layers. For example, the use of the developed model will allow one to obtain comparable selective layer thicknesses assessed from SEM investigation of membrane morphology and from gas transport experiments.

## Figures and Tables

**Table 1:** Membrane samples under investigation.

Designation	Description
Stamp 1	PE / PAN porous support
Stamp 1-2	PE / PAN / PDMS gutter layer
Stamp 1-3	PE / PAN / PDMS / PolyActive^TM^ separation layer
Stamp 2	PE / PAN porous support
Stamp 2-2	PE / PAN / PDMS gutter layer
Stamp 2-3	PE / PAN / PDMS / PolyActive^TM^ separation layer

**Table 2:** Gas transport parameters of PDMS and PolyActive™ determined from time-lag experiments.

	$P_{i}\;(\mathrm{at}\;30\;{}^{\circ}C)$ (10^−^^15^·mol·m·m^−^^2^·s^−^^1^·Pa^−^^1^) ^1^	*α* (*i*/N_2_)	$P_{\;\infty ,i}^{0}$ (10^−^^15^·mol·m·m^−^^2^·s^−^^1^·Pa^−^^1^) ^1^	$E_{\mathrm{act},\;}i^{2}$ (kJ·mol^−1^)
**PDMS of gutter layer**				
H_2_	127.0	2.8	33,424	14.0
CH_4_	135.3	3.0	5567	9.4
N_2_	45.1	1.0	8822	13.2
O_2_	101.2	2.2	6067	10.3
CO_2_	507.8	11.3	862	1.3
**PolyActive^TM^**				
H_2_	5.9	5.2	730,721	29.7
CH_4_	3.5	3.1	854,055	31.1
N_2_	1.1	1.0	607,510	33.2
O_2_	2.9	2.6	538,272	30.7
CO_2_	60.5	53.2	39,320	16.4

^1^ To obtain the permeability in Barrer (10^−10^·cm^3^·(STP)·cm·cm^−2^·s^−1^·cmHg^−1^), the value has to be multiplied by 2.99 × 10^15^. ^2^ determined in the temperature range 20 °C to 80 °C.

**Table 3:** Numerical evaluation of the gutter layer thickness determined from results of the gas transport experiments (temperature 30 °C and feed pressure 50 kPa).

Varied Parameters	Scenario			
	1	2	3	4
**Stamp No.**	**1-2**	**1-2**	**1-2**	**2-2**
*ε/τ*	0.056	0.056	0.056	0.099
*d_pore_* (nm)	133	133	133	119
*δ_PS_* (µm)	30	23	40	30
**Gas**	**Thickness of gutter layer *δ_G_* (nm)**			
H_2_	138	140	135	145
CH_4_	130	166	161	147
N_2_	136	136	135	154
O_2_	149	149	145	155
CO_2_	149	160	n.a.	160
**Average *δ_G_* (nm)**	140	150	115	150
**SD**	7	13	65	6

**Figure A3:**
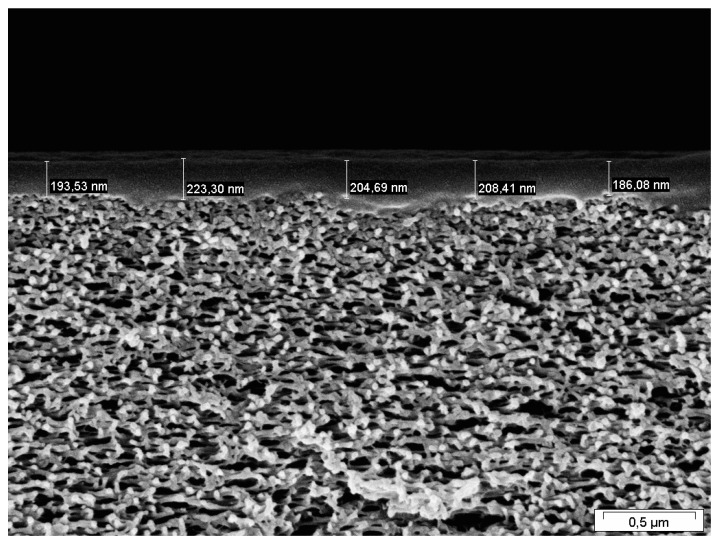
SEM micrograph of TFCM with gutter layer (PDMS) and selective layer (PolyActive^TM^). Stamp 1-3.

## Nomenclature

*Symbols*	
*A*	Cross sectional membrane area, m^2^
*d_pore_*	Pore diameter, m
*E_act_*	Activation energy of permeability, J/mol
*f*	Fugacity, Pa
*L*	Permeance, mol∙m/(m^2^∙s∙Pa)
*m*_0_	Swelling factor at zero temperature, Pa^−1^
*m_T_*	Factor of temperature dependency of the swelling, K^−1^
*M*	Molecular weight, kg/mol
$\overset{\cdot }{n}$	Flow rate, mol/s
${\overset{\cdot }{n}}^{”}$	Molar flux through the membrane divided by area, mol/(m^2^∙s)
*p*	Pressure, Pa
*P*	Permeability coefficient, mol∙m/(m^2^∙s∙Pa)
*R*	Gas constant, 8.314 Pa∙m^3^/(mol∙K)
*t*	Time, s
T	Temperature, K
*V*	Volume, m^3^
$\overset{\cdot }{V}$	Volumetric flow rate, m^3^/s
*Greek symbols*	
*_∆α_*	Thermal expansion coefficient, K^−1^
δ	Thickness of layer *j*, m
*ε*	Fractional void volume of porous medium
*η*	Dynamic viscosity of the gas, Pa∙s
*τ*	Tortuosity factor
*Subscripts*	
*a*	Average
*act*	Activation
*F*	Feed
*G*	Gutter layer
*i*	Component *i*
*j*	Layer *j*
*P*	Permeate
*pore*	Pore
*PS*	Porous support
*S*	Selective layer
*t*	Total
*T*	Temperature
*W*	Pore wall
∞	Temperature → ∞
*Superscripts*	
∙	Time derivative
´´	Area derivative
*0*	Pressure → 0

## References

[1] Henis J.M., Tripodi M.K. (1980). Multicomponent Membranes for Gas Separations. U.S. Patent.

[2] Fouda A., Chen Y., Bai J., Matsuura T. (1991). Wheatstone Bridge Model for the Laminated Polydimethylsiloxane Polyethersulfone Membrane for Gas Separation. J. Membr. Sci..

[3] Shilton S.J., Bell G., Ferguson J. (1996). The deduction of fine structural details of gas separation hollow fibre membranes using resistance modelling of gas permeation. Polymer.

[4] Shieh J.J., Chung T.S., Paul D.R. (1999). Study on multi-layer composite hollow fiber membranes for gas separation. Chem. Eng. Sci..

[5] Peng F.B., Liu J.Q., Li J.T. (2003). Analysis of the gas transport performance through PDMS/PS composite membranes using the resistances-in-series model. J. Membr. Sci..

[6] Selyanchyn R., Ariyoshi M., Fujikawa S. (2018). Thickness Effect on CO_2_/N_2_ Separation in Double Layer Pebax-1657^®^/PDMS Membranes. Membranes.

[7] Sakaguchi T., Hu Y., Masuda T. (2017). Substituted Polyacetylenes. Membrane Materials for Gas and Vapor Separation.

[8] Fritsch D., Bengtson G., Carta M., McKeown N.B. (2011). Synthesis and Gas Permeation Properties of Spirobischromane-Based Polymers of Intrinsic Microporosity. Macromol. Chem. Phys..

[9] Khan M.M., Filiz V., Bengtson G., Shishatskiy S., Rahman M.M., Lillepaerg J., Abetz V. (2013). Enhanced gas permeability by fabricating mixed matrix membranes of functionalized multiwalled carbon nanotubes and polymers of intrinsic microporosity (PIM). J. Membr. Sci..

[10] Brinkmann T., Lilleparg J., Notzke H., Pohlmann J., Shishatskiy S., Wind J., Wolff T. (2017). Development of CO2 Selective Poly(Ethylene Oxide)-Based Membranes: From Laboratory to Pilot Plant Scale. Engineering.

[11] Tena A., Rangou S., Shishatskiy S., Filiz V., Abetz V. (2016). Claisen thermally rearranged (CTR) polymers. Sci. Adv..

[12] Wijmans J.G., Hao P.J. (2015). Influence of the porous support on diffusion in composite membranes. J. Membr. Sci..

[13] Koester S., Lolsberg J., Lutz L., Marten D., Wessling M. (2016). On individual resistances of selective skin, porous support and diffusion boundary layer in water vapor permeation. J. Membr. Sci..

[14] Kattula M., Ponnuru K., Zhu L., Jia W., Lin H., Furlani E.P. (2015). Designing ultrathin film composite membranes: The impact of a gutter layer. Sci. Rep..

[15] Pauls J.R., Fritsch D., Klassen T., Peinemann K.-V. (2012). Gas permeation measurement under defined humidity via constant volume/variable pressure method. J. Membr. Sci..

[16] Mason E.A., Malinauskas A.P. (1983). Gas Transport in Porous Media: The Dusty-Gas Model.

[17] Kipp S. (2010). Charakterierung CO2-Selektiver Membranen zur Biogasaufbereitung.

[18] Lilleparg J., Georgopanos P., Shishatskiy S. (2014). Stability of blended polymeric materials for CO_2_ separation. J. Membr. Sci..

[19] Scharnagl N., Buschatz H. (2001). Polyacrylonitrile (PAN) membranes for ultra- and microfiltration. Desalination.

[20] Abetz V., Brinkmann T., Dijkstra M., Ebert K., Fritsch D., Ohlrogge K., Paul D., Peinemann K.V., Nunes S.P., Scharnagl N. (2006). Developments in membrane research: From material via process design to industrial application. Adv. Eng. Mater..

[21] Peter J., Peinemann K.V. (2009). Multilayer composite membranes for gas separation based on crosslinked PTMSP gutter layer and partially crosslinked Matrimid (R) 5218 selective layer. J. Membr. Sci..

[22] Albo J., Hagiwara H., Yanagishita H., Ito K., Tsuru T. (2014). Structural Characterization of Thin-Film Polyamide Reverse Osmosis Membranes. Ind. Eng. Chem. Res..

[23] Cabasso I., Lundy K.A. (1986). Method of Making Membranes for Gas Separation and the Composite Membranes. U.S. Patent.

[24] Lillepärg J., Georgopanos P., Emmler T., Shishatskiy S. (2016). Effect of the reactive amino and glycidyl ether terminated polyethylene oxide additives on the gas transport properties of Pebax^®^ bulk and thin film composite membranes. RSC Adv..

[25] Yave W., Car A., Funari S.S., Nunes S.P., Peinemann K.-V. (2010). CO_2_-Philic Polymer Membrane with Extremely High Separation Performance. Macromolecules.

[26] Nistor C., Shishatskiy S., Popa M., Nunes S.P. (2009). CO_2_ Selective Membranes Based on Epoxy Silane. Rev. Roum. Chim..

[27] Dai Z., Ansaloni L., Deng L. (2016). Recent advances in multi-layer composite polymeric membranes for CO_2_ separation: A review. Green Energy Environ..

[28] Krishna R., Wesselingh J.A. (1997). Review article number 50—The Maxwell-Stefan approach to mass transfer. Chem. Eng. Sci..

[29] Epstein N. (1989). On Tortuosity and the Tortuosity Factor in Flow and Diffusion through Porous-Media. Chem. Eng. Sci..

[30] Stern S.A., Fang S.M., Frisch H.L. (1972). Effect of pressure on gas permeability coefficients. A new application of “free volume” theory. J. Polym. Sci. Part A Polym. Phys..

[31] Webb S.W. (2006). Gas transport mechanisms. Gas Transport in Porous Media.

[32] Brinkmann T., Naderipour C., Pohlmann J., Wind J., Wolff T., Esche E., Müller D., Wozny G., Hoting B. (2015). Pilot scale investigations of the removal of carbon dioxide from hydrocarbon gas streams using poly (ethylene oxide)–poly (butylene terephthalate) PolyActive™) thin film composite membranes. J. Membr. Sci..

[33] Berean K., Ou J.Z., Nour M., Latham K., McSweeney C., Paull D., Halim A., Kentish S., Doherty C.M., Hill A.J. (2014). The effect of crosslinking temperature on the permeability of PDMS membranes: Evidence of extraordinary CO_2_ and CH4 gas permeation. Sep. Purif. Technol..

[34] Merkel T.C., Bondar V., Nagai K., Freeman B.D. (1999). Hydrocarbon and perfluorocarbon gas sorption in poly(dimethylsiloxane), poly(1-trimethylsilyl-1-propyne), and copolymers of tetrafluoroethylene and 2,2-bis(trifluoromethyl)-4,5-difluoro-1,3-dioxole. Macromolecules.

[35] Pinnau I., He Z.J. (2004). Pure- and mixed-gas permeation properties of polydimethylsiloxane for hydrocarbon/methane and hydrocarbon/hydrogen separation. J. Membr. Sci..

[36] Flichy N.M.B., Kazarian S.G., Lawrence C.J., Briscoe B.J. (2002). An ATR−IR Study of Poly (Dimethylsiloxane) under High-Pressure Carbon Dioxide: Simultaneous Measurement of Sorption and Swelling. J. Phys. Chem. B.

[37] Sirard S.M., Green P.F., Johnston K.P. (2001). Spectroscopic ellipsometry investigation of the swelling of poly(dimethylsiloxane) thin films with high pressure carbon dioxide. J. Phys. Chem. B.

[38] Machado D.R., Hasson D., Semiat R. (2000). Effect of solvent properties on permeate flow through nanofiltration membranes—Part II. Transport model. J. Membr. Sci..

[39] Ohlrogge K., Wind J., Brinkmann T. (2010). Membranes for Recovery of Volatile Organic Compounds. Comprehensive Membrane Science and Engineering.

[40] Matyka M., Khalili A., Koza Z. (2008). Tortuosity-porosity relation in porous media flow. Phys. Rev. E Stat. Nonlinear Soft Matter Phys..

[41] Pisani L. (2011). Simple Expression for the Tortuosity of Porous Media. Transp. Porous Media.

[42] Volkenandt T., Muller E., Gerthsen D. (2014). Sample thickness determination by scanning transmission electron microscopy at low electron energies. Microsc. Microanal..

[43] Van Krevelen D.W., te Nijenhuis K. (2009). Chapter 13—Mechanical Properties of Solid Polymers. Properties of Polymers.

[44] Zhang Y.X., Chen Y., Yan M.F., Chen F.L. (2015). New formulas for the tortuosity factor of electrochemically conducting channels. Electrochem. Commun..

[45] Robeson L.M., Smith Z.P., Freeman B.D., Paul D.R. (2014). Contributions of diffusion and solubility selectivity to the upper bound analysis for glassy gas separation membranes. J. Membr. Sci..

[46] Coefficient of Linear Thermal Expansion (CLTE): Formula & Values. https://omnexus.specialchem.com/polymer-properties/properties/coefficient-of-linear-thermal-expansion#values.

[47] Pochan D.J., Lin E.K., Satija S.K., Wu W.-L. (2001). Thermal Expansion of Supported Thin Polymer Films:  A Direct Comparison of Free Surface vs. Total Confinement. Macromolecules.

